# Genomic analysis and assessment of pathogenic (toxicogenic) potential of *Staphylococcus haemolyticus* and *Bacillus paranthracis* consortia isolated from bovine mastitis in Russia

**DOI:** 10.1038/s41598-023-45643-w

**Published:** 2023-10-30

**Authors:** Sergei Sokolov, Fedor Brovko, Alexander Solonin, Daria Nikanova, Ksenia Fursova, Olga Artyemieva, Evgenia Kolodina, Anatoly Sorokin, Margarita Shchannikova, Timur Dzhelyadin, Artem Ermakov, Khanafy Boziev, Natalia Zinovieva

**Affiliations:** 1 Laboratory of Microbiology, L.K. Ernst Federal Science Center for Animal Husbandry, Dubrovitsy, 142132 Russia; 2 Laboratory of Immunochemistry, Shemyakin and Ovchinnikov Institute of Bioorganic Chemistry of the Russian Academy of Sciences, Pushchino, 142290 Russia; 3 Laboratory of Plasmid Biology, Federal Research Center “Pushchino Scientific Center for Biological Researches”, G.K. Skryabin Institute of Biochemistry & Physiology of Microorganisms of the Russian Academy of Sciences, Pushchino, 142290 Russia; 4 Laboratory of Cell Genome Functioning Mechanisms, Federal Research Center “Pushchino Scientific Center for Biological Researches”, Institute of Cell Biophysics of the Russian Academy of Sciences, Pushchino, 142290 Russia

**Keywords:** Microbial genetics, Bacterial genes

## Abstract

Three stable microbial consortia, each composed of *Bacillus* *paranthracis* and *Staphylococcus* *haemolyticus* strains, were isolated from milk of cows diagnosed with mastitis in three geographically remote regions of Russia. The composition of these consortia remained stable following multiple passages on culture media. Apparently, this stability is due to the structure of the microbial biofilms formed by the communities. The virulence of the consortia depended on the *B. paranthracis* strains. It seems plausible that the ability of the consortia to cause mastitis in cattle was affected by mutations of the *cytK* gene of *B. paranthracis*.

## Introduction

Mastitis, inflammation of the mammary gland, is a common disease in cattle. It is related to the presence of pathogenic microorganisms, the most common of them being *E. coli* and various *Streptococcus* and *Staphylococcus* species^1^. Non-aureus staphylococci are pathogenic microorganisms frequently isolated in mammary gland infections^2–4^. Another group of microorganisms, which are less frequently isolated from milk but represent a considerable interest, are pathogenic bacilli^5^. The fact that pathogenic bacilli are capable of sporulation, which allows them to survive thermal treatment of milk and dairy products, further emphasizes their significance. The development of associations composed of different microbial species and their adaptation to their environments are currently increasingly attracting researchers' interest. In the present work, we have for the first time described consortia of *Staphylococcus haemolyticus* and *Bacillus paranthracis* isolated from the milk of cows diagnosed with mastitis in three geographically remote regions of Russia.

## Results

### General genomic characteristics

**Figure 1:**
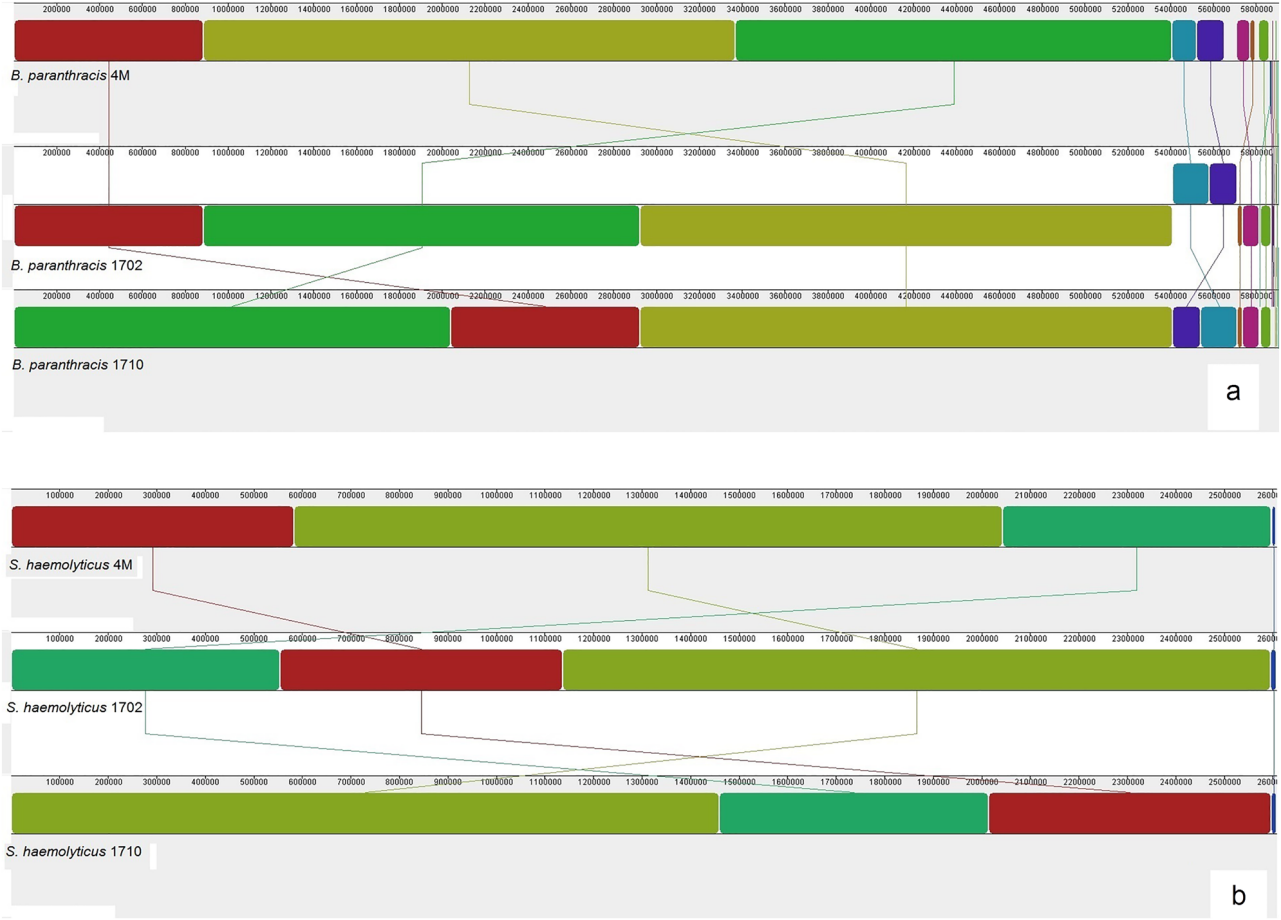
Mauve whole-genome alignments of *B. paranthracis* (**a**) and *S. haemolyticus* (**b**) strains.

Each of the three studied bacterial consortia (4M, 1702, and 1710) was composed of two pathogenic strains. Genome quality estimation with CheckM showed that all genomes were of high quality (> 98% completeness and < 0.5% contamination). The Genome Taxonomy Database tool kit (GTDB-tk) was used to classify the bacterial genomes. GTDB-tk analysis classified one of strains in each consortium as a member of the Bacillus_A group, namely *Bacillus paranthracis*. The other strain in each consortium was *Staphylococcus haemolyticus* based on the taxonomic classification defined by topology and ANI. *B. paranthracis* strains exhibited a high level of similarity among different consortia, and so did the *S. haemolyticus* strains (Fig. 1). Each of the *B. paranthracis* strains possessed ten replicons, including one chromosome and nine plasmids ranging in size from 3124 bp to more than 300 kb. Five larger plasmids apparently had the theta-type replication, whereas the smaller plasmids were of the RCR type. The *S. haemolyticus* strains contained four replicons: one chromosome and three small plasmids of 6539, 3048, and 2362 bp (Table 1). Genetic features of plasmids found in *B. paranthracis* and *S. haemolyticus* strains are listed in Supplementary Data.

**Table 1:** Genome size of *B. paranthracis* and *S. haemolyticus* strains.

	*B. paranthracis* 4M	*S. haemolyticus* 4M	*B. paranthracis* 1702	*S. haemolyticus* 1702	*B. paranthracis* 1710	*S. haemolyticus* 1710
Chromosome	5,408,718	2,596,308	5,407,047	2,595,786	5,408,561	2,595,916
Plasmid 1	303,605	6539	301,943	6539	303,603	6539
Plasmid 2	101,096	3048	101,096	3048	101,795	3048
Plasmid 3	55,506	2362	55,506	2362	55,506	2362
Plasmid 4	16,314		16,314		16,314	
Plasmid 5	7636		7635		7635	
Plasmid 6	5235		5235		5235	
Plasmid 7	3897		3899		3897	
Plasmid 8	3562		3562		3562	
Plasmid 9	3124		3124		3124	

To find out in how much the strains composing the consortia differed genetically, pairwise comparison of the *B. paranthracis* genomes, as well as of the *S. haemolyticus* genomes, was performed using the ANI calculator. The results of this comparison are shown in Table 2. It can be seen that none of the strains studied was identical to some other strain. Most likely, these consortia originated from a common ancestor consortium and have been evolving independently. The characteristics of the three *B. paranthracis* genomes were absolutely identical, and so were the characteristics of the three *S. haemolyticus* genomes; they are listed in Table 3.

**Table 2:** Pairwise comparison of genomes by ANI calculator.

ANI	*B. paranthracis* 4M	*B. paranthracis* 1702	*B. paranthracis* 1710	*S. haemolyticus* 4M	*S. haemolyticus* 1702	*S. haemolyticus* 1710
*B. paranthracis* 4M		99.93	99.90			
*B. paranthracis* 1702	99.93		99.91			
*B. paranthracis* 1710	99.90	99.91				
*S. haemolyticus* 4M					99.89	99.92
*S. haemolyticus* 1702				99.89		99.92
*S. haemolyticus* 1710				99.92	99.92

**Table 3:** Genomic features of *B. paranthracis* and *S. haemolyticus* strains.

Replicon	Genomic objects	CDS	fCDS	tRNA	rRNA	misc_RNA	tmRNA	% GC
*B. paranthracis*								
Chromosome	5984	5694	16	104	39	131	0	35.52
Plasmid 1	366	354	2	0	0	10	0	33.29
Plasmid 2	120	120	0	0	0	0	0	33.74
Plasmid 3	91	91	0	0	0	0	0	35.86
Plasmid 4	31	30	0	0	0	1	0	32.30
Plasmid 5	10	9	0	0	0	1	0	32.19
Plasmid 6	9	9	0	0	0	0	0	34.10
Plasmid 7	5	4	0	0	0	1	0	33.95
Plasmid 8	4	3	0	0	0	1	0	35.54
Plasmid 9	4	3	0	0	0	1	0	32.84
* S. haemolyticus*								
Chromosome	2732	2567	8	59	19	79	0	32.97
Plasmid 1	5	5	0	0	0	0	0	30.95
Plasmid 2	2	2	0	0	0	0	0	29.30
Plasmid 3	2	2	0	0	0	0	0	31.13

fCDS: pseudogenes; tRNA: transfer RNA; rRNA, ribosomal RNA; misc_RNA: miscellaneous RNA; tmRNA: transfer-messenger RNA.

### Putative virulence genes

**Figure 2:**
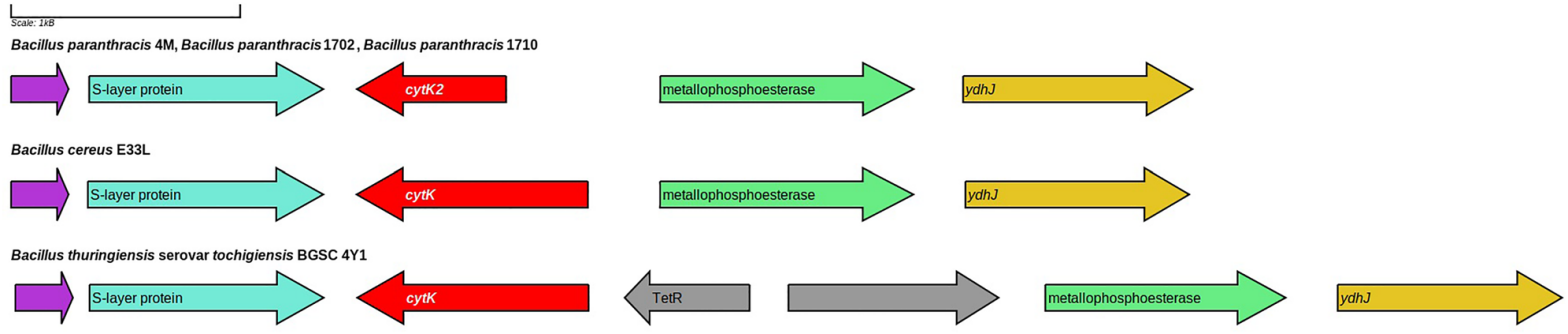
Comparison between the neighborhoods of the *cytK* gene in the *B. paranthracis*, *B. cereus* and *B. thuringiensis* genomes.

**Figure 3:**
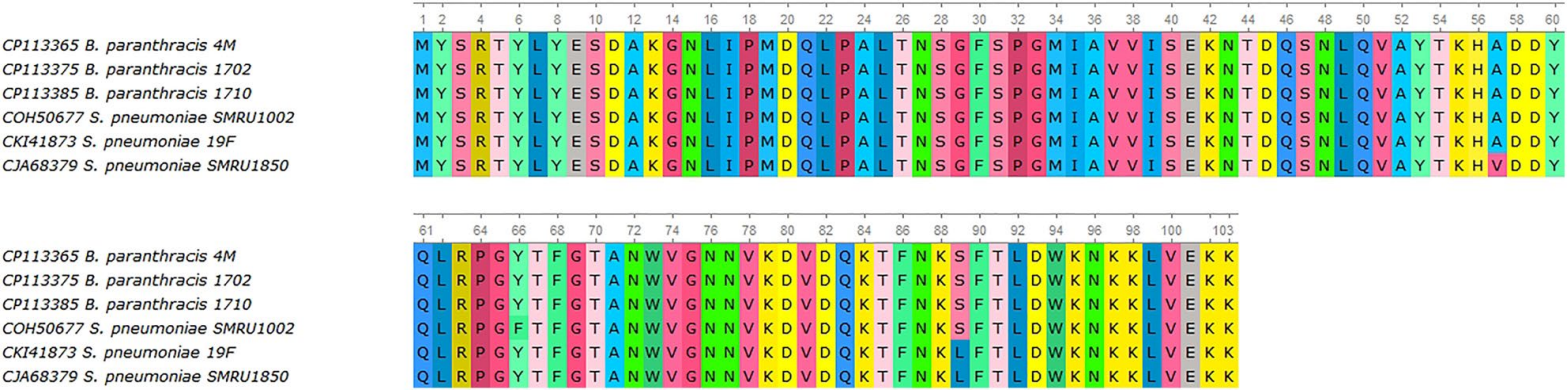
Multiple alignments of truncated CytK-2 and subunit C of hemolysin gamma amino acid sequences.

The isolated *B. paranthracis* strains were found to possess several putative virulence genes. The genes *nheA*, *nheB*, and *nheC* encode a pore-forming toxin composed of a cytolytic protein NheA and two associated protein components NheB and NheC, which enhance the biological activity of the cytolytic protein. There were also three homologous reading frames encoding a secreted metalloprotease InhA, which can cleave multiple proteins of the host organism cells. The multiple alignment and the evolutionary tree of the three *inaA* orthologs are presented in Supplementary Materials, Fig. S1. All *B. paranthracis* strains possessed two copies of the gene encoding thiol-activated cytolysin ALO, which belongs to the family of cholesterol-dependent cytolysins. ALO is a pore-forming toxin that requires the presence of cholesterol in the membrane for pore formation; to date, the mechanism of this process is not fully understood. In addition, the *B. paranthracis* genomes included a gene cluster composed of the genes *hblA1*, *hblA2*, and *hblB*, which encodes a component of pore-forming hemolysin BL. However,the most interesting finding in *B. paranthracis* strains concerned the gene of the pore-forming toxin cytK-2. In all three strains, the sequence of this gene carried a point mutation, an T → A transition at position 357 that produced a TAA stop codon interrupting the reading frame. As a result, instead of the full-size CytK-2 (336 amino acids), these cells probably synthesize its truncated variant of 103 amino acids, which rather resembles leukotoxin LukDv (Fig. 2). The multiple alignment of *cytK2* nucleotide sequence of *B. paranthracis* 4M, *B. paranthracis* 1710, *B. paranthracis* 1702, *B. cereus* E33L, and *B. thuringiensis* BGSC 4Y1 is presented in Supplementary Materials, Fig. S2. In addition, this truncated protein exhibits 99% homology to subunit C of hemolysin gamma from *Streptococcus pneumoniae* (GenBank Sequence IDs CKI41873, COH50677, and CJA68379) (Fig. 3).

The *S. haemolyticus* strains lacked genes of pore-forming toxins but possessed several genes responsible for prevention of phagocytosis. These were type 8 capsule genes: *capA*, *capB*, *capC*, *capD*, and *capP*. These genes are also involved in formation of biofilms.

### Biofilm formation

In addition to the genes *capA*, *capB*, *capC*, *capD*, and *capP* mentioned above, the isolated *S. haemolyticus* strains were found to possess the *icaC* gene, which is usually a part of the *ica* operon of intercellular adhesion required for biofilm formation. However, the other genes of this operon, *icaADB*, were not detected in the *S. haemolyticus* strains studied. In addition, the *S. haemolyticus* strains possessed the genes of the adapter proteins MecA and MecB, which play a central role in the process of biofilm formation.

The *B. paranthracis* genomes included the *eps1* cluster comprising 17 open reading frames (Supplementary Materials, Fig. S3); these strains lacked the *eps2* cluster. The genomes of *B. paranthracis* also included the genes of the multifunctional regulators *papR*, *plcR*, and *sinR*, which are of significant importance both for the expression of virulence factors and for biofilm formation. In addition, the genomes of *B. paranthracis* had other genes involved in biofilm formation: *sipW* and *tasA*; furthermore, *tasA* was represented by three orthologs. The first of them was located remote from *sipW*, upstream from a gene encoding a serine peptidase of the family S8. Two other *tasA* orthologs were located immediately downstream from *sipW* and directly upstream from *sinR* and *inaA*_2.

### Antibiotic resistance genes

The *B. paranthracis* strains possessed two chromosomal antibiotic resistance genes. One of them, *vmlR*, encodes an ATPase that binds to the ribosome and provides resistance to virginiamycin and lincomycin by the target protection mechanism. The other gene, *fosB*, encodes a magnesium-dependent thiol transferase that degrades fosfomycin.

The chromosome of the *S. haemolyticus* strains featured two resistance genes: *fusC* is responsible for inactivation of fusidic acid; *mgrA* ensures active efflux of a number of antibiotics, such as ciprofloxacin, methicillin, and tetracycline. Furthermore, the 6539-bp plasmid of *S. haemolyticus* carried two further resistance genes. One of them, *aacA–aphD*, encodes an aminoglycoside acetyltransferase and provides resistance to aminoglycoside antibiotics (e.g., neomycin, gentamicin, and kanamycin). The other gene was initially described as *tetB* in *Geobacillus stearothermophilus*; it is responsible for tetracycline efflux.

### Secondary metabolites

The *B. paranthracis* strains contained two non-ribosomal peptide synthetases responsible for the synthesis of bacillibactin and fengycin, respectively. The *S. haemolyticus* strains were capable of synthesizing staphyloferrin.

Summary of genes for the virulence, biofilm formation, and antibiotic resistance in *B. paranthracis* and *S. haemolyticus* strains is presented in Table 4.

**Table 4:** Summary of genes for the virulence, biofilm formation, and antibiotic resistance presented in each strain.

	Geographical Region I	Geographical Region II	Geographical Region III	Geographical Region I	Geographical Region II	Geographical Region III
	*B. paranthracis* 4M	*B. paranthracis* 1702	*B. paranthracis* 1710	*S. haemolyticus* 4M	*S. haemolyticus* 1702	*S. haemolyticus* 1710
I. Virulence genes						
* nheA/B/C*	+	+	+	–	–	–
* inhA*	+	+	+	–	–	–
* hblA*	+	+	+	–	–	–
* cytK2*	+	+	+	–	–	–
II. Biofilm genes						
* icaC*	–	–	–	+	+	+
* eps*	+	+	+	–	–	–
* papR*	+	+	+	–	–	–
* plcR*	+	+	+	–	–	–
* sipW*	+	+	+	–	–	–
* tasA*	+	+	+	–	–	–
III. Antibiotic resistance genes						
* vmlR*	+	+	+	–	–	–
* fosB*	+	+	+	–	–	–
* fusC*	–	–	–	+	+	+
* mgrA*	–	–	–	+	+	+
* aacA–aphD*	–	–	–	+	+	+
* tetB*	–	–	–	+	+	+

## Discussion

Mastitis is a common and economically important disease of cattle. In this work, we used the megasequencing technology to analyze the bacterial communities of the infected milk samples and identify the presence of genes that can participate both in the pathogenic process and in overcoming the host's immune response. It was found that milk samples collected from cows with mastitis consistently contained consortia of two gram-positive bacteria, *S. haemolyticus* and *B. paranthracis*. The best-known pathogenic agent associated with cattle mastitis is *Staphylococcus aureus*, a gram-positive coccus that also causes other clinical infections. It is also recognized as a pathogen provoking food poisoning outbreaks. The pathogenicity of *S. aureus* is due to a broad range of virulence factors, including hemolysins, leukocidins, enterotoxins, and secretion systems of numerous toxins^6,7^.

While *S. aureus* has been described as a major pathogen causing cattle mastitis, coagulase-negative staphylococci (CNS) are also increasingly recognized worldwide as etiologic agents associated with intramammary infections (IMI). However, clinical and pathogenetic significance of their detection in milk sample cultures is still debated. Some researchers believe that CNS are true mastitis-causing agents with major virulence factors, a high level of antimicrobial resistance, and the ability to cause chronic infections. At the same time, others consider them secondary pathogens of cattle.

Bacterial pathogens have to survive, proliferate, and spread within the host; they must be able to adapt to an extremely hostile environment by responding to the existing immune barriers. The interaction between a host and a pathogen should be viewed not as a static phenomenon, but as an arms race in which each competitor tries to act as effectively as possible^8^. As a consequence, pathogens have developed extremely sophisticated strategies to overcome the host's immune response. In particular, depending on the lifestyle of various microbial pathogens, phagocytosis as such can represent not only an obstacle but also an opportunity for their propagation: some of them employ numerous intricate strategies to enter phagocyte cells and survive in them, whereas other bacteria have developed mechanisms to prevent or evade phagocytosis. For successful infection, pathogens must overcome or avoid the activity of the host's immune system cells. Infection with virulent bacilli is characterized by bacterial proliferation in spite of inflammation at the infection site^9^. This implies that these bacteria have developed means to counteract inflammatory cells and thus the host immune system.

We performed a comparative analysis of the pathogenicity factors of the other consortium member *B. paranthracis*, which can attack epithelial cells, as well as overcome and/or suppress the host's immune response. Taxonomically, *B. paranthracis* is very close to *Bacillus cereus*, a gram-positive spore-forming bacterium that provokes food poisoning and severe opportunistic infections^10,11^. *B. cereus* is present in soil and in food products; it contaminates human skin and nearly all surfaces in hospital environments. It is the second most common cause of mass food-related infection outbreaks after *S. aureus*^12^. Therefore, *B. cereus *sensu lato is of particular interest in terms of food safety and public health because of its ability to spoil food and cause disease due to synthesis of various toxins^13^. In this study, we determined the presence of pathogenicity factors in the identified consortia. All *B. paranthracis* strains present in the consortia possessed a *cytK*-2 gene^14^ with a mutation disrupting the reading frame. The protein encoded by this new reading frame has a high level of homology to LukDv, a pore-forming toxin protein described in *S. pneumoniae*. This protein can form pores in the membranes of eukaryotic cells, unbalancing the partial pressure inside the host cells, which results in cell lysis, release of the nutrients into the environment, and ultimately in the death of these cells^15^. The novel protein is encoded by a gene with a high homology to *cytK*, which suggests a possibility of horizontal transfer of this gene among various gram-positive microorganisms that are in a physical contact.

The second best-studied genetic determinant of a pore-forming toxin in *B. cereus *sensu lato is the *hbl* gene cluster present in all *B. paranthracis* strains described in this work. Hbl-dependent pore formation requires all Hbl components, which can individually bind to erythrocytes and form a membrane attack complex that ultimately provokes cell lysis^16^. The presence of the genes encoding PlcR and its inducer PapR in all strains described in our work also serves for positive control of the expression of Hbl-encoding genes. Furthermore, the expression of the gene that encodes the protein highly homologous to LukDv apparently remained under the characteristic positive transcriptional control of the PlcR system^17^ as described for the wild-type *cytK*-2 gene.

*B. cereus* cells can survive in the host organism and cause infection in spite of attracting phagocytes. The genome of *B. cereus* includes over 350 genes that encode exoproteins; among them, there are at least 50 genes of proteases with several putative pathogenetic functions^18^. At all stages of infection with pathogenic microorganisms, proteases are key virulence factors. They contribute to colonization of the host by degrading the extracellular matrix of host tissues, including collage and elastin. Together with the cytolytic activity of pore-forming toxins, this protease-mediated degradation promotes bacterial proliferation by providing nutrients, interfering with the host's immune response, damaging the protective endothelium, and disrupting the epithelial barriers^19^. Among exoproteases of *B. cereus*, there are two zinc-dependent proteases, InhA1 and NprA, which were quantitatively determined in a study of several exoproteomes^20–22^. InhA1 plays a central role in the virulence of *B. cereus *sensu lato due to its interactions with both bacterial and host proteins in the course of infection. InhA1 and NprA both feature zinc-binding motifs and include amino acid residues of the active catalytic center common for metalloproteases (HEXXH). InhA1 is lethal when injected into the hemolymph of insects and is capable of cleaving antibacterial peptides, such as cecropin and attacin^23^. InhA1 also determines the ability of *B. cereus* cells to evade host macrophages^24^. The *B. paranthracis* strains described herein possess genes of three homologous proteins: InhA1, InhA2, and InhA3. NprA enables bacteria to escape from macrophages and therefore represents a key element required to combat the host's immune system. Mutants with inactivated *nprA* or *inhA1* genes cannot escape undamaged if captured by macrophages. NprA degrades host cell components, which can explain the role this proteins plays in the consortium during the escape from macrophages. InhA1-mediated cleavage of NprA is a key step in enhancing NprA activity. Indeed, an active C-terminal domain of NprA suffices to stimulate the release of bacteria from host macrophages after phagocytosis^25^.

Bacteria of the consortium are constantly exposed to a flow of milk and therefore require the ability to form biofilms. In the biofilm state, they adhere to the udder wall and acquire resistance to stress factors and most antibiotics, while continuing to secrete various pathogenicity factors^26^. Protection provided by a biofilm enables the bacteria to resist not only the host's defense mechanisms but also the standard antibiotic therapy. In staphylococci, biofilm formation involves the genes of the intercellular adhesion operon (*ica*), *icaADBC*, responsible for the synthesis of polysaccharide intercellular adhesin (PIA), the principal component of the exopolysaccharide matrix surrounding bacterial cells within biofilms. Most biofilms found in natural environments are composed of several bacterial species. *B. cereus *sensu lato is no exception. It is frequently observed in associations with other microorganisms, when biofilms can be described as cooperative consortia where each partner contributes to stability and development of the community^27^.

Similarly to *cytK*^28^, individual genes involved in biofilm formation in *B. cereus* are regulated by the global transcriptional regulator PlcR together with the oligopeptide encoded by *papR*, since quorum sensing is critically important not only for toxin expression levels but also for biofilm formation in gram-positive microorganisms. In addition, bacterial adhesion to surfaces, intercellular interactions, cell aggregation, and biofilm formation in *B. cereus* involve genes of the *eps2* cluster, whereas the *eps1* gene cluster apparently participates in some sort of social motility of these bacteria. That is, the *eps2* cluster is more important for adhesion to epithelial cells^29^.

In addition, the expression of enterotoxin genes is affected by the phase transition regulator SinR. SinR and PlcR regulate biofilm formation in an interactive manner. SinR was shown to control biofilm formation and swimming motility in *B. thuringiensis*^30^. Interestingly, only a small subpopulation of cells in the biofilms expressed *hbl*, which depended on the expression of *sinI*^30^. Two SinR recognition sites were detected upstream from the *hbl* operon, and one more SinR recognition site was identified upstream from the *nhe* operon^31^. It should be mentioned that the *spo0A*–*sinI*–*sinR* regulatory chain in *B. cereus* AR156 was also shown to be involved in biofilm formation, in cell differentiation, and in host–pathogen interaction^32^.

## Methods

### Strains, culturing conditions, and genomic DNA extraction

**Figure 4:**
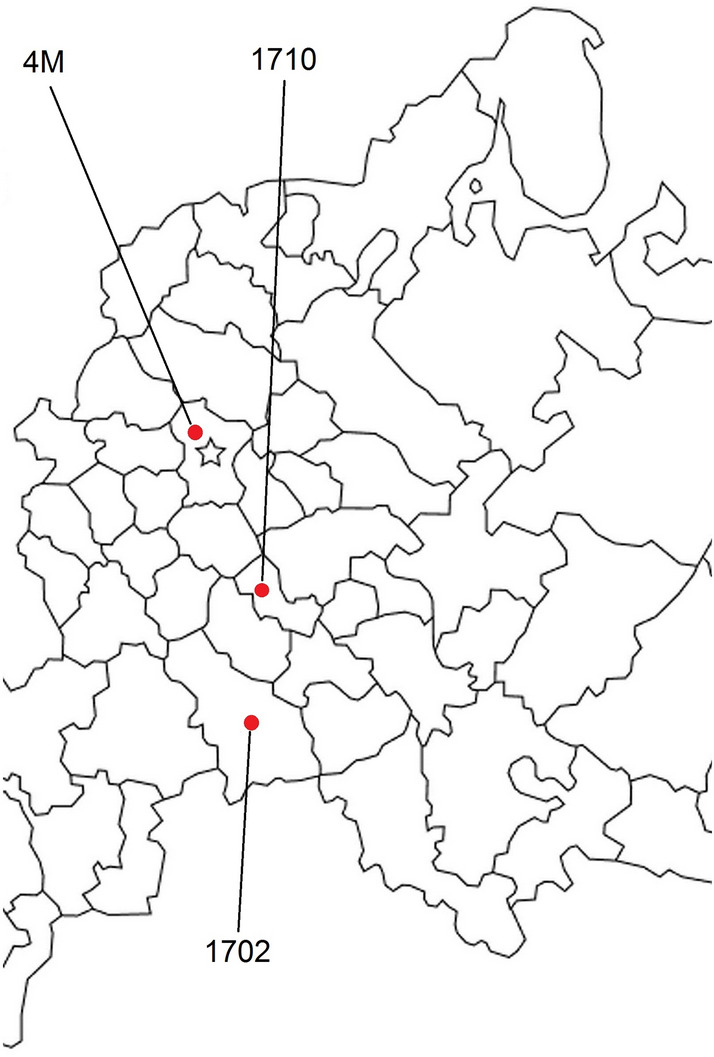
Geographical location of sample collection sites on the map of Western Russia.

Microbial consortium 4M was isolated from milk of black-motley cows in the Moscow region. Microbial consortium 1702 was isolated from milk of Simmental cows in the Saratov region. Microbial consortium 1710 was isolated from milk of black-motley Holstein hybrid cows in the Republic of Mordovia. On the map, the sites of sample collection form a triangle with sides of approximately 600, 400, and 300 km (Fig. 4). Based on the results of veterinary and laboratory control, all animals included in the study were diagnosed with the clinical form of mastitis. To obtain pure cultures, milk samples were first inoculated as enrichment cultures in Salt Meat Broth, Azide Dextrose Broth, and Tryptone Soya Broth (HiMedia Laboratories Pvt. Ltd., India) and subsequently transferred onto differential diagnostic media: Baird Parker Agar (HiMedia Laboratories) and Azide Blood Agar Pronadisa (Conda, Spain). For all cultures studied, preliminary microscopy of individual colonies from Baird Parker Agar showed cells of different size and shape (cocci and bacilli) and of the same positive Gram staining. To isolate individual strains and obtain uniform colonies, a series of five passages on the same differential diagnosis medium was performed. After each passage, microscopic analysis revealed the same two types of cells in the field of view.

Cells from a pure, fresh 50 mL liquid culture from each consortium were harvested by centrifugation at 15,000×*g* for 3 min and washed three times with sterile Milli Q water under sterile conditions. Total genomic DNA was extracted using the GeneJET Genomic DNA Purification Kit (Thermo Fisher Scientific, Bremen, Germany) following the manufacturer’s instructions. Quality and quantity of the extracted DNA was determined using QubitTM fluorometric quantitation and NanoDrop 2000 (both Thermo Fisher Scientific, Bremen, Germany).

### Genome sequencing, assembly and annotation

The genome of both strains in each consortium was sequenced on an Illumina MiSeq platform with a read length of 300 bp (paired end) and Oxford Nanopore MinION. The genome from each member of consortia was assembled using Unicycler from both short and long sequencing reads^33^. Genome quality estimation was determined with CheckM. Genome circularization was checked manually using BLAST. Genome annotation was performed with Prokka^34^. Protein coding genes were classified based on the annotation into Cluster of Orthologous Groups (COG) functional categories with the automatic classification COG tool at MicroScope platform^35^. The data for this study have been deposited in the GenBank under project number PRJNA865942 with accession numbers CP102659-CP102662, and CP113365-CP113402.

### Genomes comparison

Classification of the genomes was determined according to the Genome Taxonomy Database (GTDB) using the GTDB-tool kit (GTDB-tk) v.1.1.0 integrated in the MicroScope web-based service^35^. GTDB-tk provides a taxonomic classification of bacterial and archaeal genomes based on the combination of the GTDB reference tree, the relative evolutionary divergence and the ANI value against reference genomes^36^. CJ Bioscience's online Average Nucleotide Identity (ANI) calculator was used to compare two prokaryotic genome sequences^37^. Whole-genome alignments were performed by Mauve v. 20150226^38^.

### Ethics statement

The animal study was reviewed and approved by L.K. Ernst Federal Science Center for Animal Husbandry Bioethics Committee, protocol number 2021-3/4. All methods were carried out in accordance with the approved guidelines and relevant regulations. All methods are reported in accordance with ARRIVE guidelines.

### Supplementary Information

Supplementary Information.

Supplementary Figures.

## Data Availability

The data for this study have been deposited in the GenBank under project number PRJNA865942 with accession numbers CP113365-CP113374 for *B. paranthracis* 4M; CP102659-CP102662 for *S. haemolyticus* 4M; CP113375-CP113384 for *B. paranthracis* 1702; CP113395-CP113398 for *S. haemolyticus* 1702;CP113385-CP113394 for *B. paranthracis* 1710, and CP113399-CP113402 for *S. haemolyticus* 1710.
