# Supplementary material for: Genomic analysis and assessment of pathogenic (toxicogenic) potential of Staphylococcus haemolyticus and Bacillus paranthracis consortia isolated from bovine mastitis in Russia
Source: Sci Rep. 2023 Oct 30;13:18646. doi: 10.1038/s41598-023-45643-w (PMC10616132; doi:10.1038/s41598-023-45643-w)
Supplement: Supplementary file 2 — Supplementary Figures. [file 41598_2023_45643_MOESM2_ESM.pdf]

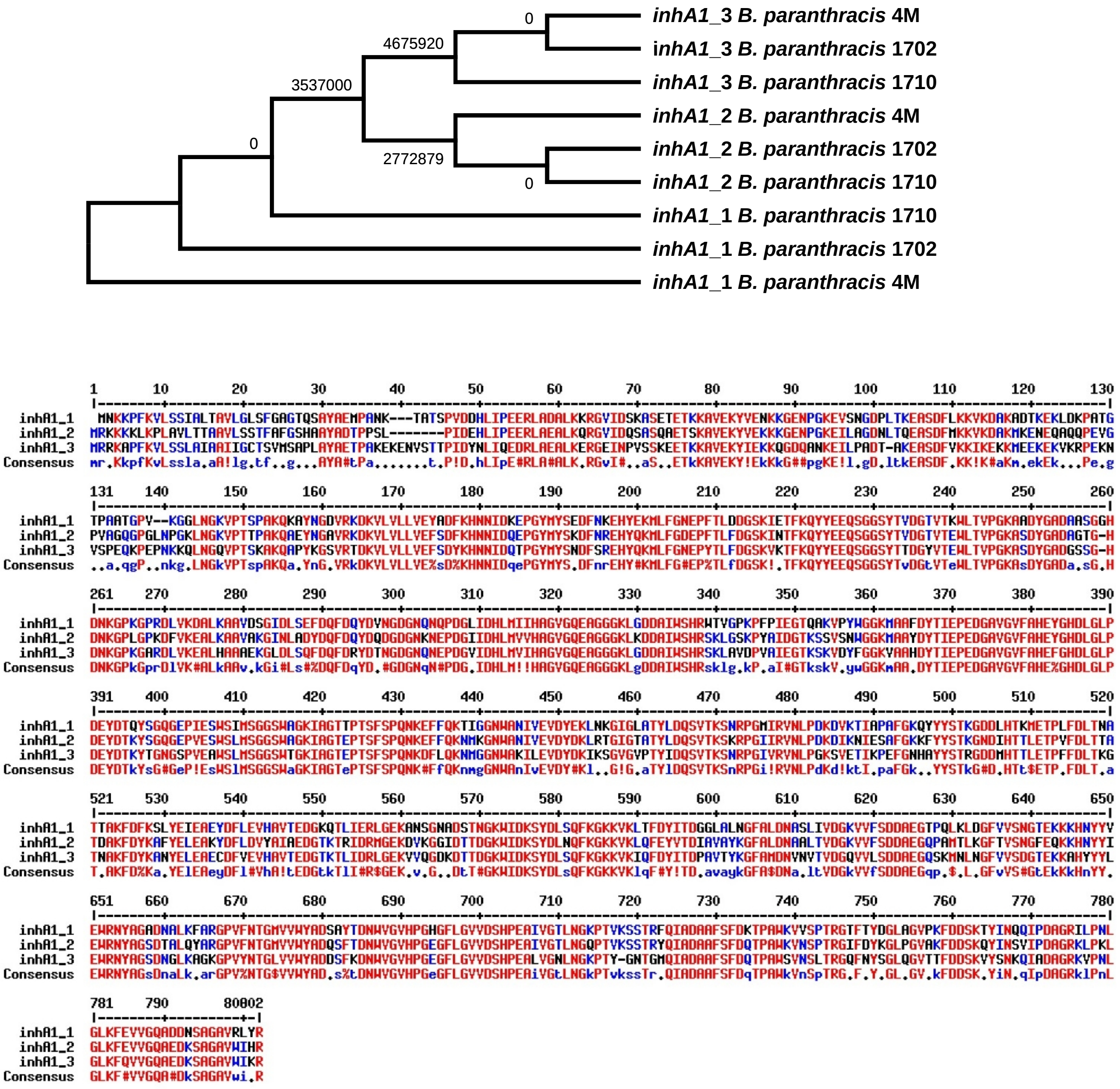

Fig. s1. Distance tree and multiple alignment of the *B. paranthracis inhA1* genes.

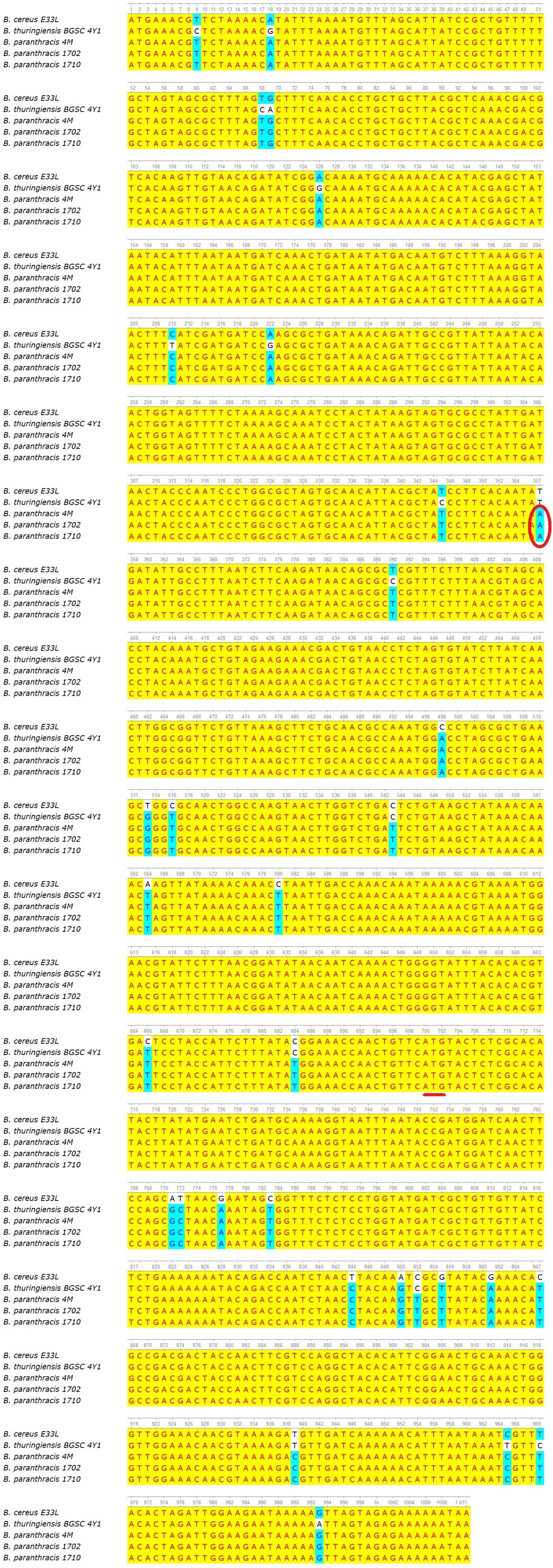

Fig. s2. Multiple nucleotide alignment of *cytK* genes. The point mutation leading to a stop-codon appearance is in red oval. Putative start codon for the truncated *cytK-2* is underlined.

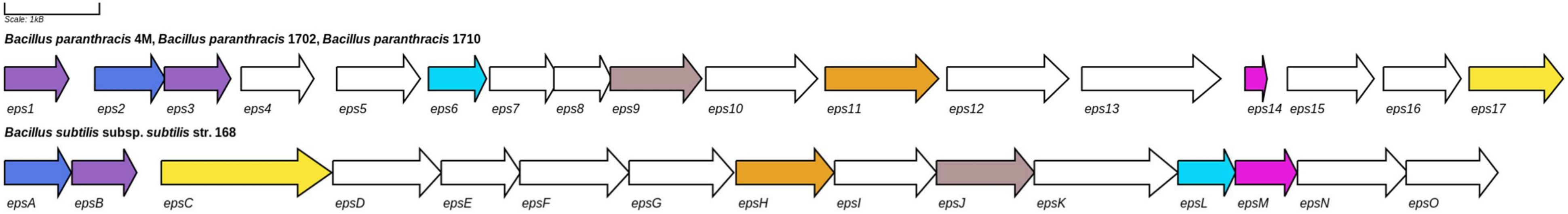

eps1	Tyrosine-protein kinase YwqD
eps2	putative capsular polysaccharide biosynthesis protein YwqC
eps3	Tyrosine-protein kinase YwqD
eps4	Tyrosine-protein phosphatase YwqE
eps5	UTP--glucose-1-phosphate uridylyltransferase
eps6	putative sugar transferase EpsL
eps7	hypothetical protein (PHP domain-containing protein)
eps8	Adenine phosphoribosyltransferase
eps9	putative glycosyltransferase EpsJ
eps10	hypothetical protein (O-antigen ligase family protein)
eps11	hypothetical protein (glycosyltransferase family 4 protein)
eps12	UDP-N-acetyl-D-mannosamine dehydrogenase
eps13	lipid II flippase MurJ
eps14	2,3,4,5-tetrahydropyridine-2,6-dicarboxylate N-acetyltransferase
eps15	Polyisoprenyl-teichoic acid--peptidoglycan teichoic acid transferase TagU
eps16	hypothetical protein (SGNH/GDSL hydrolase family protein, EPSX protein)
eps17	UDP-glucose 4-epimerase

Fig. s3. Comparison of the genetic structures of the *B. paranthracis eps1* region and the *B. subtilis epsA-O* operon. Orthologous genes are in the same colors. Automatic annotation of the genes in the *eps1* region is shown.
